# Biguanide therapy for diabetes and cancer & a novel methyltransferase that regulates energy expenditure and adiposity and is elevated in many cancers

**DOI:** 10.1186/2049-3002-2-S1-O25

**Published:** 2014-05-28

**Authors:** Barbara B Kahn, Daniel Kraus, Qin Yang

**Affiliations:** 1 Beth Israel Deaconess Medical Center and Harvard Medical School, Boston, MA, USA

## 

Compounds containing guanidine-related molecules have been used since medieval times to relieve symptoms of diabetes mellitus. Guanidine itself was too toxic but two linked guanidine rings (biguanides) were useful and generally safer. For 20 years, phenformin was used before adverse effects led to removal from the US and European markets. Metformin continues to be widely prescribed for diabetes treatment and is associated with improved survival compared to other diabetes drugs. With the current interest in using the more potent phenformin for cancer therapy, it is timely to review the risks, benefits, and potential contraindications.

The adipose cell is as an endocrine organ in addition to its role in energy storage. Adipocytes secrete hormones, cytokines and other factors that influence energy balance, glucose homeostasis, insulin sensitivity and vascular biology. In humans with obesity and type 2 diabetes, expression of the Glut4 glucose transporter is down-regulated selectively in adipocytes resulting in systemic insulin resistance. Knocking out Glut4 selectively in adipocytes in mice increases the risk of diabetes while adipose-specific Glut4 overexpression confers enhanced glucose tolerance in spite of increased adiposity. To understand how adipocytes regulate glucose homeostasis and insulin sensitivity, we performed DNA microarray analysis of adipose tissue from mice with adipose-specific knockdown or overexpression of Glut4. The most highly reciprocally regulated gene is nicotinamide N-methyltransferase (*Nnmt*). NNMT methylates nicotinamide (vitamin B3) using S-adenosylmethionine (SAM) as a methyl donor. Nicotinamide is a precursor for NAD^+^, an important cofactor linking cellular redox states with energy metabolism. NNMT expression is increased in adipose tissue and liver of obese and diabetic mice. Furthermore, NNMT knockdown in adipose tissue and liver using anti-sense oligonucleotides in mice on a high fat diet, protects against diet-induced obesity by augmenting cellular energy expenditure. This involves a novel mechanism which could provide new strategies for prevention and treatment of obesity and type 2 diabetes. Since NNMT is also elevated in many cancers, NNMT knockdown could be beneficial for cancer prevention and/or treatment.

